# The mutation of *ent*-kaurene synthase, a key enzyme involved in gibberellin biosynthesis, confers a non-heading phenotype to Chinese cabbage (*Brassica rapa* L. ssp. *pekinensis*)

**DOI:** 10.1038/s41438-020-00399-6

**Published:** 2020-11-01

**Authors:** Yue Gao, Shengnan Huang, Gaoyang Qu, Wei Fu, Meidi Zhang, Zhiyong Liu, Hui Feng

**Affiliations:** Liaoning Key Laboratory of Genetics and Breeding for Cruciferous Vegetable Crops, College of Horticulture, Shenyang Agricultural University, 110866 Shenyang, China

**Keywords:** Plant breeding, Plant breeding

## Abstract

The presence of a leafy head is a vital agronomic trait that facilitates the evaluation of the yield and quality of Chinese cabbage. A non-heading mutant (*nhm1*) was identified in an ethyl methanesulfonate mutagenesis population of the heading Chinese cabbage double haploid line FT. Segregation analysis revealed that a single recessive gene, *Brnhm1*, controlled the mutant phenotype. Using MutMap, Kompetitive allele-specific PCR, and cloning analyses, we demonstrated that *BraA07g042410.3C*, which encodes an *ent*-kaurene synthase involved in the gibberellin biosynthesis pathway, is the *nhm1* mutant candidate gene. A single-nucleotide mutation (C to T) in the fourth exon of *BraA07g042410.3C* caused an amino acid substitution from histidine to tyrosine. Compared to that of the wild-type FT, *BraA07g042410.3C* in the leaves of the *nhm1* mutant had lower levels of expression. In addition, gibberellin contents were lower in the mutant than in the wild type, and the mutant plant phenotype could be restored to that of the wild type after exogenous GA_3_ treatment. These results indicate that *BraA07g042410.3C* caused the non-heading mutation. This is the first study to demonstrate a relationship between gibberellin content in the leaves and leafy head formation in Chinese cabbage. These findings facilitate the understanding of the mechanisms underlying leafy head development in Chinese cabbage.

## Introduction

Chinese cabbage (*Brassica rapa* L. ssp. *pekinensis*) is a widely cultivated and economically important vegetable species in China and Southeast Asia. The presence of a leafy head is an essential morphological trait for evaluating the yield and quality of Chinese cabbage. Leafy heads of Chinese cabbage are rich in vitamins, dietary fiber, and nutrients^1,2^.

**Fig. 1:**
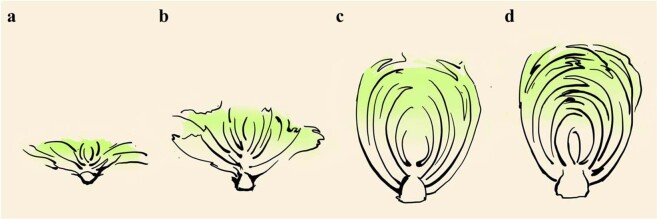
Four stages of leafy head formation in Chinese cabbage. **a** Seedling stage: primary leaves grow. **b** Rosette stage: rosette leaves change to fold upward from being flat and provide photosynthetic products for leafy head formation. **c** Folding stage: the folding leaves are curved inward. **d** Heading stage: heading leaves surrounding the shoot apexes form a compact head. From the rosette stage to the heading stage, low-temperature induction, low light intensity, a low carbohydrate/nitrogen ratio, uneven distribution of phytohormones, antagonism of adaxial and abaxial polar genes, and the signaling pathways of multiple hormone, include auxin, GA, ethylene, jasmonic acid (JA), abscisic acid (ABA), brassinolide (BR), cytokinin (CK), and salicylic acid (SA), may play critical roles in controlling leafy head development

The formation of leafy heads in Chinese cabbage is biologically complex and usually spans four stages of development: the seedling stage, rosette stage, folding stage, and heading stage^3^ (Fig. 1). Numerous factors, such as auxin concentrations, light intensity, temperature, and the carbohydrate-to-nitrogen ratio, may influence this process^4^. Several genes associated with the formation of Chinese cabbage leafy heads have been reported in previous studies. For instance, indoleacetic acid (IAA) induces transcriptional expression of the *BcpLH* (*LEAFY HEADS*) gene, indirectly promoting leafy head formation^1^. Mao et al. ^5^ suggested that the expression levels of *BrpTCP* (*TEOSINTE BRANCHED 1*, *cycloidea*, and *PCF transcription factor*) may affect the shape of leafy heads. Wang et al. ^6^ revealed that *BrpSPL9* (*SQUAMOSA PROMOTER BINDING*-*LIKE 9*) genes could shorten the seedling and rosette stages to make the formation of leafy heads occur earlier. Moreover, Yu et al. ^7^ revealed the *BrAN3* (*ANGUSTIFOLIA 3*) gene controls the formation of leafy heads. Other previous studies have also shown that multiple genes control leafy head formation^8,9^. Therefore, further investigation of the genes associated with leafy head formation could enhance our understanding of leafy head development.

Auxins are the most important hormones that affect the formation of leafy head in Chinese cabbage. Li^10^ suggested that changes in the concentrations of auxin in the adaxial and abaxial surfaces of leaves cause the leaves to curl inwards to form a leafy head. Similarly, He et al.^3^ suggested that auxin concentrations and distribution could influence the formation of leafy heads. Using a genome convergence approach to analyze the genomes of *B. rapa* and *B. oleracea*, Cheng et al. ^2^ found that various processes, including auxin-mediated signaling, may result in similar leafy head formation in Chinese cabbage and cabbage. Although some studies have shown that the synthesis, transport, and signal transduction of phytohormones, especially auxins, regulate the formation of Chinese cabbage leafy heads, the underlying molecular mechanisms and the genetic basis of leafy head development remain unclear.

**Fig. 2:**
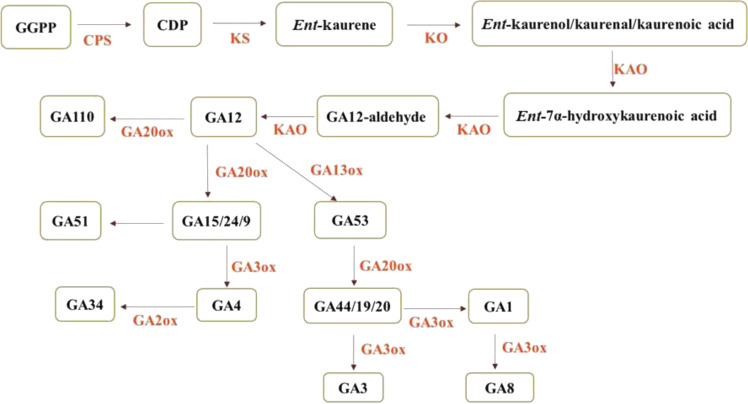
Schematic representation of the gibberellin biosynthesis pathway (refer to Binenbaum J. et al., 2018^25^)

In the present study, we identified a non-heading mutant, *nhm1*, whose mutation is controlled by a single recessive gene, from a Chinese cabbage ethyl methanesulfonate (EMS) mutagenesis population. Based on MutMap and Kompetitive Allele-Specific PCR (KASP) analyses, we demonstrate that *BraA07g042410.3C*, which encodes an *ent*-kaurene synthase (*KS*) participating in gibberellin (GA) biosynthesis, is the *nhm1* candidate gene. As a key enzyme, *KS* plays an important role in GA synthesis (Fig. 2), and its mutation should affect plant development. By determining endogenous GA contents and applying exogenous GA_3_ treatment, we demonstrated that *nhm1* was a GA-deficient mutant. In contrast to previous studies, our study is the first to report that GA contents in leaves are related to the formation of leafy heads in Chinese cabbage. These results provide novel insights that could facilitate our understanding of the mechanisms of leafy head formation in Chinese cabbage.

## Results

### Phenotypic analysis and inheritance of *nhm1*

**Fig. 3:**
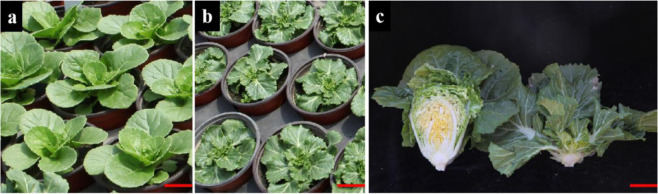
Phenotypes of the wild type FT and *nhm1* mutant. **a** Wild-type FT plants at the rosette stage. The plants are shown at 45 DAS (days after sowing). Bar = 5 cm. **b** Mutant *nhm1* plants at the rosette stage. The plants are shown at 45 DAS. Bar = 5 cm. **c** Wild-type FT (left) and *nhm1* mutant (right) plants at the heading stage. The plants are shown at 100 DAS. Bar = 10 cm

**Fig. 4:**
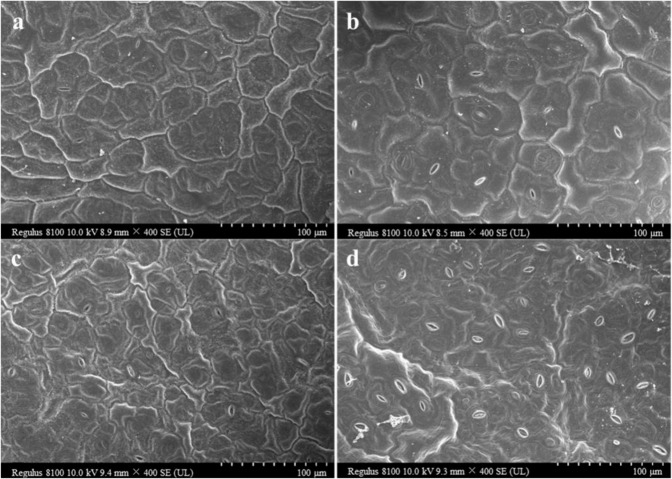
Scanning electron microscopy images of wild-type FT and *nhm1* mutant leaves. **a**, **c** Adaxial and abaxial epidermal cells of the wild type FT. **b**, **d** Adaxial and abaxial epidermal cells of the *nhm1* mutant

The leaves of the mutant exhibited geotropic growth throughout all stages of development, and leafy heads were not formed during the heading stage (Fig. 3). This is in contrast to the wild-type heading Chinese cabbage double haploid (DH) line FT, which exhibited leafy head formation. Based on analysis of scanning electron microscopy (SEM) images, there was no significant difference in the areas of the adaxial and abaxial epidermal cells between the wild-type FT and the *nhm1* mutant at the rosette stage (Fig. 4).

The *nhm1* phenotypic data are listed in Table 1. All the F_1_ plants obtained from the reciprocal cross between the wild-type FT and *nhm1* mutant showed the wild-type phenotype. The segregation ratio between the wild-type and mutant phenotypes in F_2_ plants was close to 3:1 (*χ*^2^ = 0.16). In addition, in the backcross (F_1_ × *nhm1* mutant) generation, the separation ratio was close to 1:1 (*χ*^2^ = 0.10). Therefore, we concluded that the *nhm1* mutant trait was controlled by a single recessive nuclear gene, which we named *Brnhm1*.

**Table 1:** Genetic analyses of the *nhm1* mutant.

Generations	Total plants	Mutant plants	Wild-type plants	Segregation ratio	*χ*^2^
P_1_ (FT)	50	0	50		
P_2_ (*nhm1*)	50	50	0		
F_1_ (P_1_ × P_2_)	30	0	30		
F_1_ (P_2_ × P_1_)	30	0	30		
BC_1_ (F_1_ × FT)	80	0	80		
BC_1_ (F_1_ × *nhm1*)	90	47	43	1.09:1	0.10
F_2_	400	96	304	3.17:1	0.16

### Identification of candidate genes using MutMap

Through genome resequencing of the wild-type FT, *nhm1* mutant, and the DNA pool, we obtained 135,076,750, 187,153,604, and 170,387,334 high-quality reads, respectively. Subsequently, 96.6%, 97.97%, and 97.78% of the clean reads in the wild-type FT, *nhm1*, and DNA pool, respectively, could be aligned to the reference genome.

**Fig. 5:**
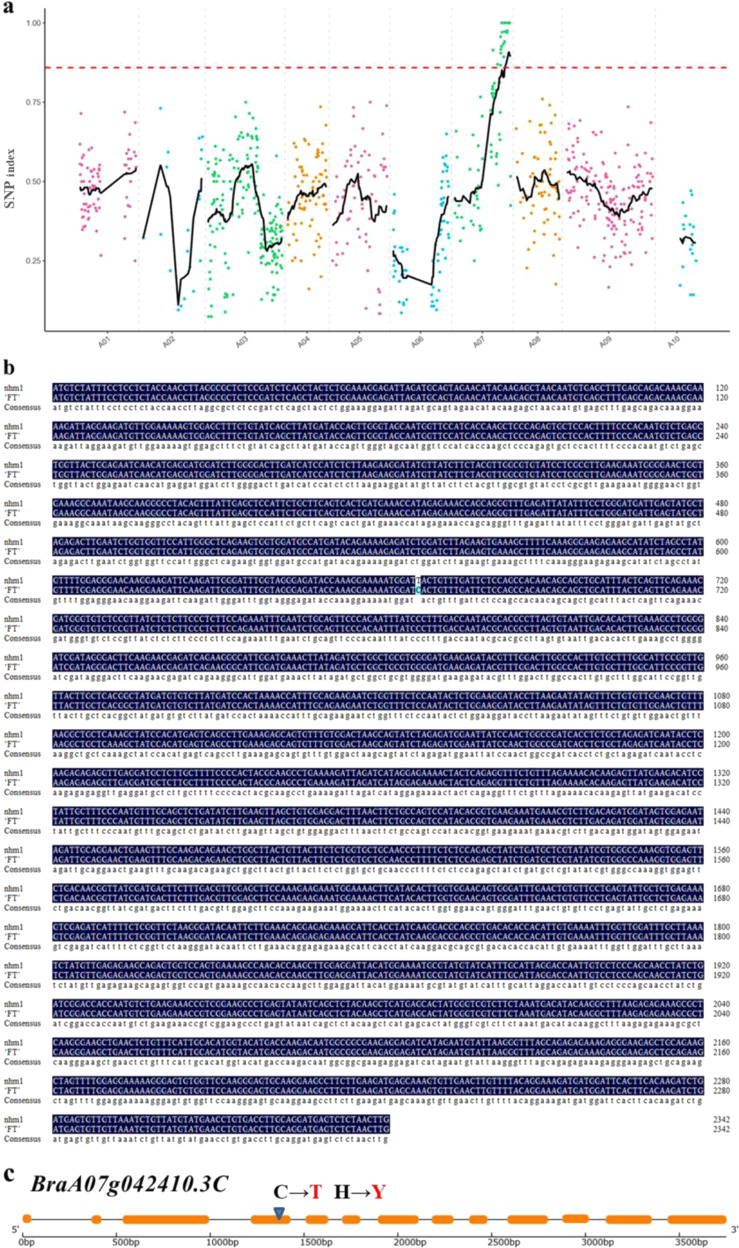
Identification of the nhm1 candidate gene. **a** SNP index plot of 10 chromosomes produced by MutMap analysis. The *x*-axis represents the positions of the 10 chromosomes; the *y*-axis represents the SNP index. The dotted pink line represents the index threshold (0.86). **b** Comparison of the *BraA07g042410.3C* coding sequence between the wild type FT and the *nhm1* mutant. **c** Structure of the predicted *BraA07g042410.3C*. The yellow boxes and lines represent exons and introns, respectively

When a single-nucleotide polymorphism (SNP) index of 0.86 was used as the threshold, we located a candidate region of 2.91 Mb (26,010,373–28,918,429) on chromosome A07 (Fig. 5a). We found two SNP mutations (SNP A07, 27,142,769; SNP A07, 28,395,204) that were localized to the exon and caused the nonsynonymous mutation (Table S1).

### SNP genotyping through KASP

To confirm the causal SNP, we used F_2_ individuals to conduct KASP genotyping analysis of two mutated SNPs. The genotypic assay showed that the SNP (A07, 28,395,204) of *BraA07g042410.3C* cosegregated with the mutant phenotype. The mutant-phenotype plants had a T:T genotype, and the wild-type-phenotype plants had C:T or C:C genotypes for this SNP (Table S2). However, a recombinant event was observed at another SNP (A07, 27,142,769); here, both the G:G and G:A genotypes were detected in the mutant-phenotype plants (Table S2). This further confirms that *BraA07g042410.3C* was the causal gene of the *nhm1* mutant. *BraA07g042410.3C* is homologous to *Arabidopsis AtKS1* (*At1g79460*) and encodes the key enzyme *KS* in the biosynthesis pathway of GA.

### Candidate gene cloning and sequence analysis

The MutMap and KASP analyses supported *BraA07g042410.3C* being the candidate gene for *nhm1*. Gene annotations showed that *BraA07g042410* was 3752 bp in length and consisted of 13 exons. We cloned the cDNA sequences of *BraA07g042410.3C* from wild type FT and the *nhm1* mutant. There was only a single base substitution at position A07 (28,395,204; C to T) in *nhm1*, which caused an amino acid to change from histidine (H) to tyrosine (Y) (Fig. 5b, c).

### Expression analysis of *BraA07g042410.3C* in *nhm1*

**Fig. 6:**
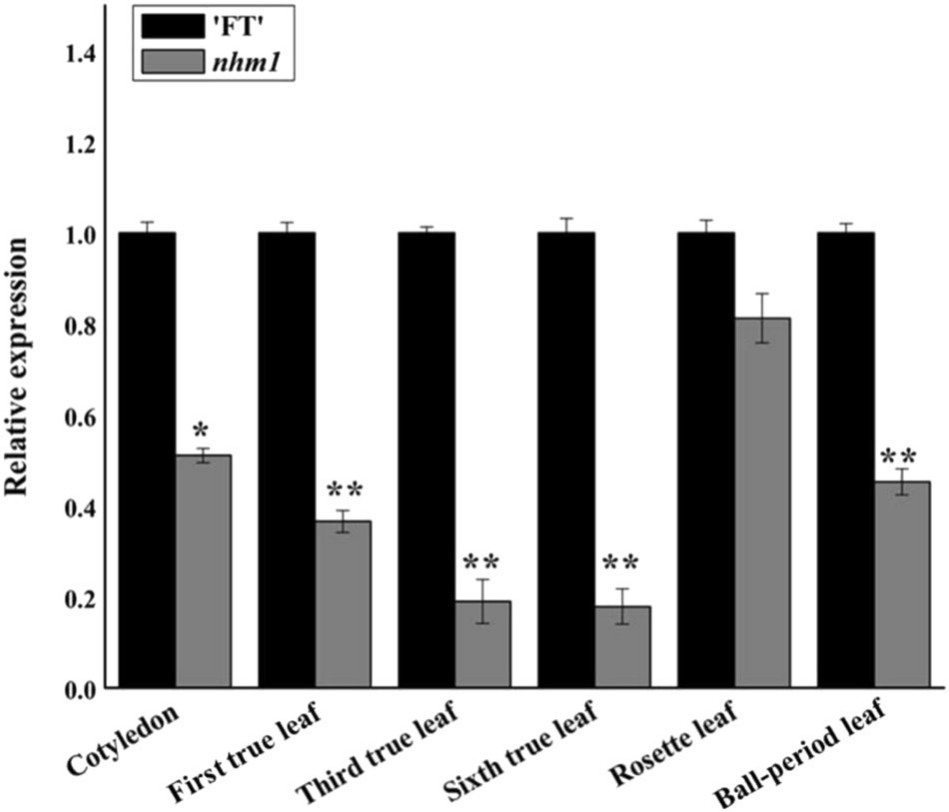
Expression levels of *BraA07g042410.3C* in the leaves of the wild-type FT and *nhm1* plants, based on qRT-PCR

We performed quantitative real-time PCR (qRT-PCR) to measure the expression level of *BraA07g042410.3C* in the leaves of the wild type FT and *nhm1* mutant. *BraA07g042410.3C* expression levels were lower in the *nhm1* mutant than in wild type FT in all four stages of leaf development (Fig. 6).

The data are the means of three replicates (±SDs). *, ** Significant differences in expression levels at *P* < 0.05 and *P* < 0.01, respectively (Student’s *t*-test).

### *nhm1* mutant plants had lower levels of GA and could be rescued by GA_3_ treatment

**Fig. 7:**
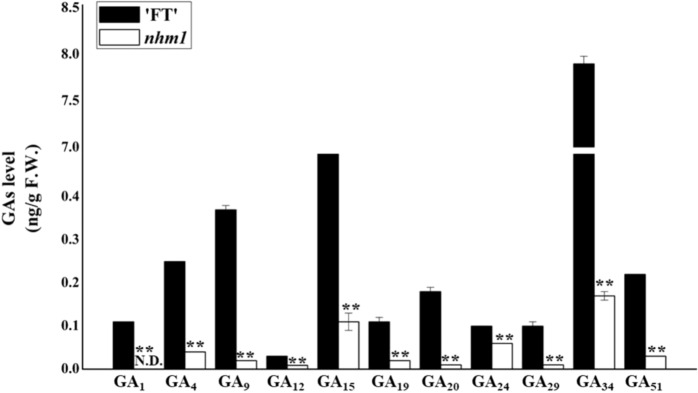
Determination of endogenous GA levels in the wild-type and mutant. The data are the means ± SDs of three trials (ng/g fresh weight). ND not detectable. **Significant differences at *P* < 0.01 (Student’s *t*-test)

**Fig. 8:**
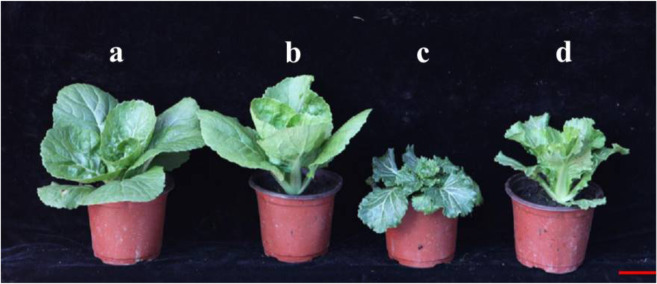
Phenotypes of the wild-type FT and *nhm1* plants in response to exogenous GA_3_ treatments. **a** Wild-type FT, **b** wild-type FT treated with GA_3_, **c** *nhm1* mutant, and **d** *nhm1* mutant plants treated with GA_3_. The plants are shown at 55 DAS. Bar = 5 cm

Bioactive GA_1_ was detected only in the wild-type plants, and the level of bioactive GA_4_ was significantly lower in the *nhm1* mutant than in the wild type. However, bioactive GA_3_ and GA_7_ were not detected. In addition, the GA content (GA_9_, GA_12_, GA_15_, GA_19_, GA_20_, GA_24_, GA_29_, GA_34_, and GA_51_) was significantly lower in the *nhm1* mutant than in the wild type (Fig. 7). Based on the differences in GA contents between the wild-type and mutant plants, we investigated the effects of exogenous GA_3_ application on the leaves of *nhm1* mutant plants. As illustrated in Fig. 8, the phenotype of the mutant plants treated with exogenous GA_3_ could be restored to wild-type phenotype.

## Discussion

In the present study, a Chinese cabbage EMS-induced non-heading mutant, *nhm1*, was identified. First, phenotypic identification revealed that the leaves of the mutant appeared to exhibit geotropic growth during the entire growth period, resulting in non-heading. Second, following MutMap and KASP analyses, a nonsynonymous mutation was detected in the fourth exon of *BraA07g042410.3C*. This gene encodes a protein with *KS* activity and thereby catalyzes the second step in the cyclization of GGPP to *ent*-kaurene in the GA biosynthesis pathway; we found that this gene harbors an SNP on A07 (at 28,395,204). Third, cloning and sequence analyses revealed that this mutant SNP involved only a single base substitution (C to T), which caused an amino acid to change from H to Y. Fourth, the *BraA07g042410.3C* expression levels were lower in the leaves of the mutant than in the leaves of the wild-type plants. Consistent with the qRT-PCR results, the GA contents in the mutant were significantly lower than those in the wild type. Last, the mutant phenotype could be rescued after exogenous GA_3_ treatment. The results indicate that the mutation in *BraA07g042410.3C*, which encodes a key GA biosynthesis enzyme, was responsible for the mutant phenotype.

GAs, which are class of diterpenoid plant hormones, play key roles in plant life cycles, such as in the promotion of cell elongation and division, hypocotyl and stem elongation, seed germination, root growth, and flowering^11,12^. To ensure normal plant growth and development, active GAs must be produced and must accumulate. However, the biosynthesis of active GAs requires various functional enzymes to catalyze different intermediates, which involves a complex and multistep process^13^. In the model plant species *Arabidopsis thaliana* and rice, the main genes that encode key enzymes in each step of the GA biosynthetic pathway have been identified^14–16^. In the GA biosynthetic pathway, copalyl pyrophosphate synthase catalyzes the cyclization of GGDP to CDP at an early step, whereas *Ent*-kaurenoic acid oxidase in the endoplasmic reticulum catalyzes the conversion of *ent*-kaurene acid to GA_12_^12,17,18^. *KS* catalyzes the second step in the cyclization of GGPP into *ent*-kaurene in the GA biosynthetic pathway. Given that *KS* the second key enzyme in the GA synthesis pathway, mutations in the gene encoding this protein considerably affect plant development. At present, the gene encoding *KS* has been cloned in pumpkin, *Arabidopsis thaliana*, stevia, lettuce and other herbs^19–22^. The *GA2* mutant of *Arabidopsis thaliana* exhibits a lack of *KS* catalytic function, and germination cannot occur without GAs^20^. Silencing of *OsKS1* in rice resulted in the emergence of semidwarf plants^23^. GA-3 oxidase (GA3ox), GA-2 oxidase (GA2ox), and GA-20 oxidase (GA20ox) play important roles in the final steps of the production of biologically active GAs^24,25^. GA_12_ is converted into GA_4_ via GA_15_, GA_24_, and GA_9_ precursors and is also converted into GA_1_ through the precursors GA_53_, GA_53_, GA_44_, GA_19_, and GA_20_ in the 13-hydroxylation pathway^26^. GA_1_, GA_3_, GA_4_ and GA_7_ are thought to function as bioactive forms in plants. In the present study, we measured the endogenous GA levels in the leaves of mutants and the wild type. As illustrated in Fig. 7, the level of bioactive GA_4_ in the mutant was significantly lower than that in the wild type. We speculate that this could be because the endogenous GA_9_, GA_12_, GA_15_, and GA_24_ levels were significantly lower in the mutant than in the wild type. The absence of bioactive GA_1_ in the *nhm1* mutant may have been due to the significantly lower endogenous GA_12_, GA_19_, GA_20_, GA_44_, and GA_53_ levels in the mutant than in the wild type. In addition, bioactive GA_3_ and GA_7_ were not detected in either the mutant or the wild type. These results indicate that the *KS* mutation may affect GA biosynthesis and that the non-heading phenotype may be caused by a lack of bioactive GAs.

Previous studies have indicated that GA is one of the most important hormones in regulating plant height. Mutations in *GA3ox* resulted in dwarf-type phenotypes of maize^27^ and rice^28^. A mutation in the *sd1* allele, which encodes *GA20ox*, in the IR8 rice variety reduced endogenous GA levels and led to a short stature^29,30^. In oilseed rape, the semidwarf mutant gene *ds-3*, which encodes a DELLA protein that negatively regulates plant height via the GA signaling pathway, was identified^31^. Li et al. ^32^ suggested that *GmDW1* encodes *KS* and was responsible for a dwarf phenotype in soybean; the phenotypes of mutant plants that were treated with exogenous GA_3_ were restored to those of the wild type. In the present study, a non-heading *nhm1* mutant was identified. All leaves of the *nhm1* mutant exhibited geotropic growth during the entire growth period, and leafy heads did not form. The mutated gene-encoded *KS* is a key enzyme in GA synthesis. We have demonstrated that *nhm1* is a GA-deficient mutant through the determination of endogenous GAs and the exogenous application of GA_3_. In addition, we have demonstrated that the mutation of *KS* is responsible for the non-heading phenotype of Chinese cabbage.

Many previous studies have suggested that leafy head formation is linked to auxins in Chinese cabbage. He et al. ^3^ reported that auxins participate in the regulation of leafy head formation. Gao et al. ^33^ conducted a whole-genome annotation and a bioinformatics analysis of the auxin transport genes *BrAUX/LAX* (*AUXIN/LIKE AUXIN RESISTANT*), *BrPIN* (*PIN-FORMED*), and *BrPGP* (*P-GLYCOPROTEIN*) in Chinese cabbage and found that they play important roles in leafy head development. The authors also observed that polar auxin transport and the uneven distribution of auxins in leaves influenced the formation of leafy heads. Li et al. ^34^ identified a non-heading mutant (*fg-1*) of Chinese cabbage and showed that auxin and ABA signaling pathways were involved in regulating the formation of the leafy head. Our study is the first to demonstrate that GA contents in the leaves are related to leafy head formation in Chinese cabbage. Although the formation of leafy heads in Chinese cabbage may involve interactions among multiple hormones, how such hormones interact and their influence on leafy head formation require further investigation.

In conclusion, a non-heading mutant of Chinese cabbage, *nhm1*, was obtained following EMS mutagenesis. We demonstrated that *BraA07g042410.3C*, which encodes *KS*, an enzyme involved in the GA biosynthesis pathway, conferred a non-heading phenotype to the plants. These findings could facilitate our understanding of the mechanisms underlying leafy head formation and provide a genetic resource for Chinese cabbage crop improvement studies and activities.

## Materials and methods

### Plant materials and mutant screening

FT is a DH line that is derived from the Chinese cabbage variety Fukuda 50 and exhibits desirable characteristics such as heat resistance, early maturity, an overlapping heading type, a compact head and a short growth period (only 45 days). In total, 7800 FT seeds were germinated at 25 °C for 12 h. The germinated seeds were subsequently immersed in 0.8% EMS and placed in a 50-turn shaker for 12 h. The treated seeds were then washed thoroughly in flowing water for 12 h and sown into trays. The surviving plants (M_0_) were self-pollinated, and the M_1_ seeds were harvested. A total of 14 stably inherited non-heading mutant (*nhm*) plants were harvested by screening the M_1_ generation and identification in the M_2_ generation. We selected a non-heading mutant, named *nhm1*, as the study material.

### Genetic analysis of *nhm1*

For genetic analysis, wild type FT (P_1_) was crossed with the *nhm1* mutant (P_2_) to generate F_1_ and F_2_ populations. Phenotypic characterization was performed for each generation (P_1_, P_2_, F_1_, F_2_), and the segregation ratios of the BC_1_ and F_2_ populations were analyzed using the chi-square (*χ*^2^) test. All plants were germinated and sown in a greenhouse at Shenyang Agricultural University.

### Scanning electron microscopy

To observe the sizes of the adaxial and abaxial epidermal cells, similar parts of the rosette leaves from the wild type FT and the *nhm1* mutant were fixed in 2.5% glutaraldehyde solution at 4 °C for 48 h, processed according to the methods of Lin et al. ^35^, and then examined under SEM (Hitachi TM3030, Japan).

### Identification of candidate genes using MutMap

The candidate gene was identified by the modified MutMap method^36^. Fifty mutant plants were selected from among the F_2_ plants, and a DNA pool was constructed by pooling equal amounts of plant leaf tissue from each plant. DNA from the wild type FT, *nhm1* mutant, and the DNA pool was extracted using a DNAsecure Plant Kit (Tiangen, Beijing, China) and was re-sequenced using a NovaSeq 6000 System sequencer (Illumina, San Diego, USA).

Raw reads, including reads with adapters, reads with more than 10% unknown bases, and low-quality reads, were filtered and removed. The clean reads were then aligned to the Brassica reference genome sequence^37^ (brassicadb.org/brad/datasets/pub/Genomes/Brassica_rapa/V3.0/) using Burrows-Wheeler Aligner (BWA)^38^. We used SAMtools to transform the alignment files into SAM/BWA files and to perform SNP calling^39^. The detection of SNPs and insertion–deletions was conducted using the GATK software toolkit^40^, and the results were analyzed using ANNOVAR^41^. Circos^42^ was used to plot variations in the genome. We determined the SNP indices for 10 Chinese cabbage chromosomes. The ΔSNP index across the chromosomes of the *B. rapa* genome was obtained using sliding-window analysis, with a 4 Mb window size and a 10 kb increment for each step. The process for SNP filtering was as follows: first, we filtered out SNPs with multiple genotypes; second, we filtered out parental heterozygous SNPs; third, we filtered out SNPs that were not typical EMS-mutagenized-type mutations (C to T or G to A); and fourth, we retained SNPs that were homozygous and those that differed between the parents.

### SNP genotyping through KASP

KASP genotyping analysis was used to detect the cosegregation of each SNP and to identify *nhm1* candidate genes. One hundred eighty-four F_2_ individual plants, including 50 plants that exhibited a mutant phenotype and 134 plants that had the normal phenotype, were used for KASP genotyping. The experiment was conducted by the Vegetable Research Center of Agriculture and Forestry Academy in Beijing. The allele-specific primers used are listed in Table S3.

### Cloning and sequencing of the candidate gene

The coding sequences of candidate genes in wild type FT and the *nhm1* mutant were amplified by full-length PCR primers (Table S4). We purified the PCR products with a Gel Extraction Kit (CWBIO, Beijing, China) and then introduced the purified products into a pGEM-T Easy Vector (Promega, USA). The products were sequenced at GENEWIZ (Suzhou, China). We analyzed the sequences using DNAMAN V6 (Lynnon BioSoft, Canada).

### Candidate gene expression analysis

The expression patterns of the *nhm1* candidate gene were analyzed via qRT-PCR. Total RNA from the cotyledon, first true leaf, third true leaf, sixth true leaf, rosette leaf, and heading leaf of both the wild-type FT plants and the *nhm1* mutant plants was extracted using TRIzol reagent (Invitrogen, USA) following the manufacturer’s instructions. First-strand cDNA was synthesized using FastQuant RT Super Mix (Tiangen). The cDNA was subsequently used as a template for qRT-PCR together with SYBR Green PCR Master Mix (TaKaRa, Dalian, China). *Actin* was used as an internal control, and we used the 2^−ΔΔCt^ method to calculate relative gene expression levels^43^. Each experiment was performed for three biological replicates, and Bio-Rad iCycler IQ5 Manager (Bio-Rad Laboratories, Inc., Hercules, CA) was used to analyze the data. The sequences of the primers used for qRT-PCR are listed in Table S5.

### Endogenous GA determination and exogenous GA_3_ treatment

Endogenous GA levels in the leaves from both the wild-type and the mutant plants were determined using liquid chromatography–tandem mass chromatography. Wild-type FT and *nhm1* mutant plants were grown under the same conditions. The third true leaves were obtained, after which the leaves were frozen directly in liquid nitrogen, ground to fine powder, and extracted with 1.0 mL of 80% methanol (v/v) at 4 °C for 12 h. The extraction and content determination of GAs (GA_1_, GA_3_, GA_4_, GA_7_, GA_8_, GA_9_, GA_12_, GA_13_, GA_15_, GA_19_, GA_20_, GA_23_, GA_24_, GA_29_, GA_34_, GA_44_, GA_51_ and GA_53_) were based on those of a method reported previously^44^, with minor modifications.

To explore the response of *nhm1* to GA, seedlings were sprayed with exogenous GA_3_ solution (500 mg/L) six times at one-day intervals once the cotyledons had fully expanded. The control plants were treated with similar volumes of distilled water. Each treatment had three replicates. GA_3_ powder (Solarbio) was first dissolved in 5 mL of ethanol and then diluted to the final concentration with distilled water.

## Supplementary information

SUPPLEMENTAL MATERIAL
